# Epidemiological profile of patients with rifampicin-resistant tuberculosis: an analysis of the Uganda National Tuberculosis Reference Laboratory Surveillance Data, 2014–2018

**DOI:** 10.1186/s13756-021-00947-2

**Published:** 2021-05-08

**Authors:** Gloria Bahizi, Robert Kaos Majwala, Stevens Kisaka, Abdunoor Nyombi, Kenneth Musisi, Benon Kwesiga, Lilian Bulage, Alex Riolexus Ario, Stavia Turyahabwe

**Affiliations:** 1 Uganda Public Health Fellowship Program, Ministry of Health, Kampala, Uganda; 2 National Tuberculosis and Leprosy Division, Ministry of Health, Kampala, Uganda; 3 Department of Epidemiology and Biostatistics, School of Public Health, College of Health Sciences, Makerere University, Kampala, Uganda; 4 National Tuberculosis Reference Laboratory, Kampala, Uganda; 5 United States Agency for International Development, Defeat TB Project, Kampala, Uganda; 6 Ministry of Health, Kampala, Uganda

**Keywords:** Tuberculosis, Rifampicin-resistance, Multi-drug resistant, Epidemiology, Uganda

## Abstract

**Background:**

Drug-resistant tuberculosis (DR-TB), including rifampicin-resistant tuberculosis (RR-TB) and multidrug-resistant tuberculosis (MDR-TB, or RR-TB with additional isoniazid resistance), presents challenges to TB control. In Uganda, the GeneXpert test provides point-of-care testing for TB and rifampicin resistance. Patients identified with RR-TB receive culture-based drug susceptibility testing (DST) to identify additional resistance, if any. There are few data on the epidemiological profiles of current DR-TB patients in Uganda. We described patients with RR-TB in Uganda and assessed the trends of RR-TB to inform TB control interventions.

**Methods:**

We identified patients with RR-TB whose samples were referred for culture and DST during 2014–2018 from routinely-generated laboratory surveillance data at the Uganda National Tuberculosis Reference Laboratory. Data on patient demographics and drug sensitivity profile of *Mycobacterium tuberculosis* isolates were abstracted. Population data were obtained from the Uganda Bureau of Statistics to calculate incidence. Descriptive epidemiology was performed, and logistic regression used to assess trends.

**Results:**

We identified 1474 patients whose mean age was 36 ± 17 years. Overall incidence was 3.8/100,000 population. Males were more affected by RR-TB than females (4.9 vs. 2.7/100,000, *p* ≤ 0.01). Geographically, Northern Uganda was the most affected region (IR = 6.9/100,000) followed by the Central region (IR = 5.01/100,000). The overall population incidence of RR-TB increased by 20% over the evaluation period (OR = 1.2; 95% CI 1.15–1.23); RR-TB in new TB cases increased by 35% (OR = 1.35; 95% CI 1.3–1.4) and by 7% in previously-treated cases (OR = 1.07; 95% CI 1.0–1.1). Of the 1474 patients with RR-TB, 923 (63%) were culture-positive of whom 670 (72%) had full DST available. Based on the DST results, 522/670 (78%) had MDR-TB.

**Conclusion:**

Between 2014 and 2018, the incidence of RR-TB increased especially among newly-diagnosed TB patients. We recommend intensified efforts and screening for early diagnosis especially among previously treated patients. Mechanisms should be in put to ensure that all patients with RR-TB obtain DST.

**Supplementary Information:**

The online version contains supplementary material available at 10.1186/s13756-021-00947-2.

## Introduction

Drug-resistant tuberculosis (DR-TB) undermines the global efforts to combat TB. Rifampicin-resistant-TB (RR-TB) defined as a *Mycobacterium tuberculosis* isolate with resistance to rifampicin detected using genotypic or phenotypic methods, with or without resistance to other first-line anti-TB drugs [1]. When TB is resistant to both rifampicin and isoniazid (the two traditional first-line drugs), it is termed multidrug-resistant TB (MDR-TB). Drug-resistant TB can be present in a primary TB infection or can develop as a complication during the course of patient treatment [2]. In 2018, 3.4% of newly-diagnosed and 18% previously-treated TB patients were estimated to have MDR-TB worldwide [3]. In Uganda, MDR-TB prevalence was estimated at 1% and 12% among new TB cases and previously treated patients respectively in 2018 [4]. Infection with MDR-TB has been associated with poorer treatment outcomes, longer treatment times, and higher treatment costs as compared to infection with drug-sensitive TB [5]. The impact is exacerbated in resource-limited settings due to the presence of other infectious diseases and limited access to well-equipped healthcare facilities [6, 7].

Tuberculosis control depends on the rapid detection and successful treatment of infectious patients, both to save lives and prevent onward transmission, especially for MDR-TB. To improve targeted diagnosis and treatment, in 2010, WHO recommended the GeneXpert MTB/RIF assay, which identifies *Mycobacterium tuberculosis* mutations associated with rifampicin with high sensitivity [8]. This test has a shorter turn-around time (about 2 h) than the gold standard method of culture (2–6 weeks) [9, 10]. In 2019, 236 of the 1500 TB diagnosis and treatment units in Uganda had GeneXpert machines [11]. At these GeneXpert sites, patients whose samples are identified as rifampicin-resistant are referred to a TB drug-resistance treatment unit.

Because nearly all RR-TB isolates also are resistant to isoniazid [12], RR-TB cases diagnosed by GeneXpert are treated using a standard regimen for MDR-TB [13]. However, RR-TB isolates may also be resistant to other drugs. To further guide management of patients with RR-TB, culture and drug susceptibility testing (DST) is recommended to identify any other anti-TB drug resistance. Patients diagnosed with RR-TB at the GeneXpert sites are referred to one of the 17 TB drug-resistance treatment units in Uganda [14], where the practice is to take a second sputum sample for culture and DST at the National Tuberculosis References Laboratory (NTRL). The DST results are then used to guide patient management [15].

Active surveillance involving active case finding through culture and DST at all levels of the health system is the most effective way to monitor the spread of resistant TB strains. However, it is resource-intensive, and many countries affected by TB are unable to implement this approach. This is why in Uganda, surveillance for drug-resistant TB is largely passive, relying on individuals presenting themselves to health care facilities [16]. For TB patients identified, contact tracing is done to identify and treat those who would have been exposed. In addition, culture and DST capacity for *Mycobacterium tuberculosis* is limited to a centralized NTRL facility. All specimens requiring these specialized tests must be referred from the GeneXpert health facilities located in rural and urban settings throughout the country to the NTRL [17]. This centralized testing requires rapid and safe transport of specimens from health facilities or lower-level laboratories to the higher-level laboratory, as well as expedient reporting of results back to clinicians [18]. However, there are challenges in specimen referral and result reporting systems which contribute to diagnostic delays in routine practice [19, 20]. Such delays have a potential to translate into under-reporting of drug resistance among TB isolates. In such situations, routinely generated surveillance data are important to monitor the effectiveness of TB control programs [21]. We described the epidemiology of rifampicin resistant TB patients and their drug resistance profiles in Uganda based on routinely generated laboratory surveillance data at the NTRL during 2014–2018.

## Methods

### Study site and design

This was a retrospective analysis of laboratory data collected at the National TB Reference Laboratory (NTRL) of Uganda from 2014 to 2018. All samples from the entire country are submitted to the NTRL for both culture and DST for *Mycobacterium tuberculosis* isolates. The NTRL is a one of the WHO approved Supra National Reference Laboratories for Africa and it is accredited by the South African National Accreditation System.

### Study population

All records of patients with RR-TB whose samples were submitted to the NTRL for culture and DST during the period 2014–2018 were included in this study. The data were retrieved from the from the Laboratory Information System (LIS) where they are entered at sample submission and testing conclusion. The LIS of the NTRL is used to track patient samples from entry into the laboratory to the point when results are dispatched to the requesting health facilities. At the drug resistance treatment sites, data are entered in the Drug-Resistant Management Information System (DRMIS) for patient management.

### Study variables and data abstraction

The primary outcome was the presence of any rifampicin resistance, in the form of mono-resistance, poly-resistance, MDR, or extensive drug resistance (XDR) in a *Mycobacterium tuberculosis* isolate. For each case-patient with RR-TB, we abstracted data on age, sex, district of residence of the patient, date of enrolment on treatment, culture results, patient categorization a new or previously treated, and the drug susceptibility profile from the LIS. Classification of drug resistance was based on the DST profiles of clinical isolates, as described by the WHO definitions and reporting framework [1, 22, 23] (Table 1). Using unique identifiers, we matched laboratory data in the LIS with information in the DRMIS to obtain the case-patient’s HIV status and history of any TB treatment. Records of patients with incomplete DST, patients without DST results, and those who were susceptible to all drugs were excluded in the classification of drug resistance type. Data on GeneXpert installations across the country over the study period were collected to compare with the numbers diagnosed with RR-TB.

**Table 1:** Definitions used for the classification of drug susceptibility profiles of patients with rifampicin-resistant tuberculosis, Uganda, 2014–2018

Variable	Definition
Rifampicin resistance (RR)	Resistance to rifampicin detected using phenotypic or genotypic methods, with or without resistance to other anti-TB drugs. It includes any resistance to rifampicin, in the form of monoresistance, polyresistance, MDR or XDR
Monoresistance	Resistance to one first-line anti-TB drug only (streptomycin, rifampicin, isoniazid, ethambutol, pyrazinamide)
Polydrug resistance	Resistance to more than one first-line anti-TB drug (other than both isoniazid and rifampicin)
Multidrug resistance (MDR)	Resistance to at least both isoniazid and rifampicin
Pre-extensively drug-resistant tuberculosis (Pre-XDR TB)	MDR-TB with resistance to fluoroquinolones (ofloxacin, levofloxacin and moxifloxacin) or a second-line injectable (amikacin, kanamycin, or capreomycin), but not both
Extensively drug resistant TB (XDR TB)	Resistance to isoniazid and rifampin plus resistance to any fluoroquinolone (ofloxacin, levofloxacin and moxifloxacin) and at least one of three injectable second-line drugs (i.e., amikacin, kanamycin, or capreomycin)

### Data management and analysis

Data were entered into EpiInfo 7.2 for analysis. Descriptive statistics of the sample by person, place, and time were calculated. Line graphs were drawn to demonstrate trends whose significance was tested using logistic regression. Quantum Geographic Information System (QGIS) software was used to show the spatial trends of RR-TB in Uganda. DR-TB incidence rates were calculated using RR-TB cases per district and individual district populations. These were displayed on choropleth maps. District population estimates were calculated based on data from the 2014 National Population and Housing Census (cite), with a national growth rate of 3% to estimate the yearly populations [24]. Data on GeneXpert machine site distribution were superimposed on the incidence rate maps to analyze the relationship between incidence and GeneXpert machine availability.

## Results

### Characteristics of patients with RR-TB

During the period under assessment, 1474 RR-TB patients, with a median age of 36 (IQR 17) years, were identified. Of these, 943 (64%) were male; 568 (38.5%) were new and 848 (57.5%) were previously-treated patients. In addition, 58 (3.9%) had an unknown / unrecorded history of treatment. Further, approximately half (687; 47%) were HIV-positive and 923 (63%) had positive cultures (Table 2).

**Table 2:** Socio-demographic and baseline clinical characteristics of patients with rifampicin resistant tuberculosis, Uganda, 2014–2018

Variable	All patients N = 1474	%
*Sex*		
Female	531	36
Male	943	64
*Age group*		
0–14	51	3.5
15–24	210	14
25–34	449	30
35–44	396	27
45–54	216	15
55–64	98	6.7
65+	54	3.7
*HIV status*		
Negative	681	46
Positive	687	47
Unknown	106	7.2
*Patient category*		
New	568	39
Previously treated	848	58
Unknown	58	3.93
*Culture result*		
Negative	531	36
Positive	923	63
Others	20	1.4

### Incidence rate of rifampicin resistant tuberculosis, Uganda, 2014–2018

The overall incidence of RR-TB was 3.8/100,000 population. Males were more affected than females (4.9 vs 2.7/100,000, *p* ≤ 0.01). Persons aged 35–44 years were most affected (IR = 12/100,000) followed by those between 25 and 34 (IR = 8.5/100,000) while those aged 0–14 years were the least affected (IR = 0.23/100,000).

### Spatial distribution of rifampicin resistant tuberculosis patients, Uganda, 2014–2018

**Fig. 1:**
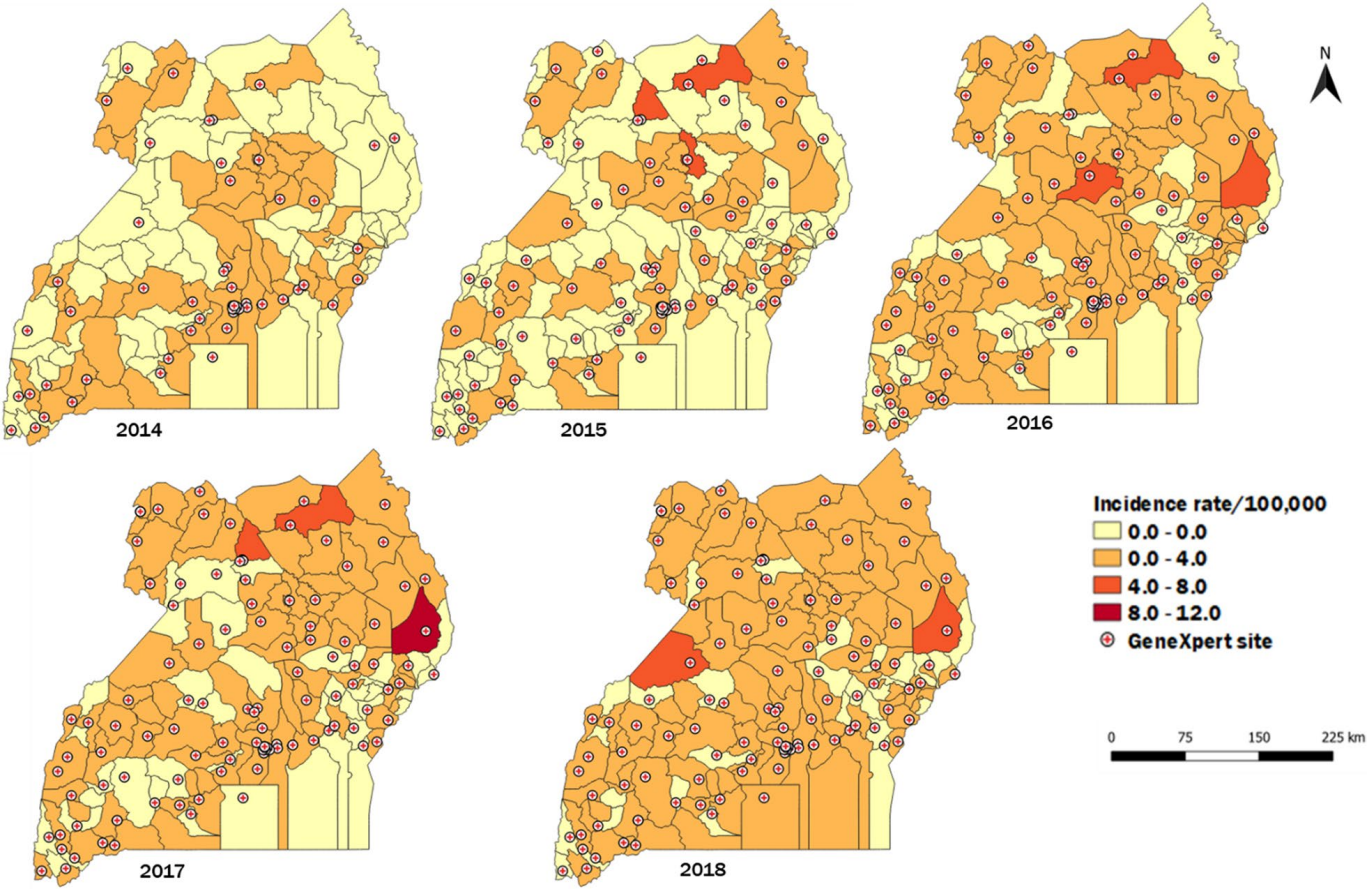
Rifampicin-resistant tuberculosis incidence per 100,000 population, Uganda, 2014–2018

District coverage with GeneXpert machines in 1500 TB diagnostic units was 4.8% in 2014, 7.4% (2015), 7.5% (2016), 9% (2017), and 17% (2018). Similarly, there were increases in RR-TB diagnosis over time (Fig. 1). The northern region of Uganda had the highest rates of rifampicin-resistant TB patients (IR = 6.9/100,000), followed by the central region (IR = 5/100,000), western region (IR = 2.7/100,000), and eastern region (IR = 2.4/100,000) (Fig. 1). The districts with the highest incidence rates in the years 2014 to 2018 are shown in Additional file 1: Table S1.

### Drug-resistance profiles of patients with RR-TB, Uganda, 2014–2018

Of the 923 culture-positive patients, 707 (77%) had their DST done. Among those with DST results, 37 (5%) had incomplete DST (first-line susceptibility testing only) and 670 (95%) had complete DST (both first and second-line). Twenty-eight (3%) of the 670 patient isolates with complete DST were susceptible to all drugs.

**Fig. 2:**
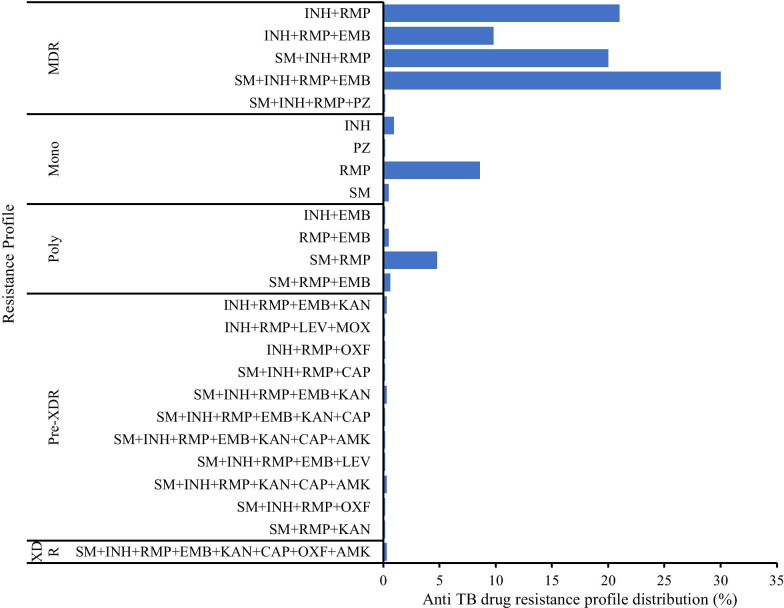
Anti-tuberculosis drug resistance profile of patients with rifampicin resistant tuberculosis, N = 642, Uganda 2014–2018. INH, Isoniazid; RMP, Rifampicin; SM, Streptomycin; EMB, Ethambutol; PZ, Pyrazinamide; KAN, Kanamycin; LEV, Levofloxacin; MOX, Moxifloxacin; OFL, Ofloxacin; CAP, Capreomycin; AMK, Amikacin

Of the 642 isolates resistant to any TB drug, multidrug resistance was the most common (522; 81%) followed by monoresistance (65; 10%), poly-resistance (39; 6%), pre-XDR (11; 1.7%), and XDR (5; 0.8%) (Fig. 2).

### Trends in incidence of rifampicin resistant tuberculosis, Uganda, 2014–2018

The incidence rate of rifampicin-resistant TB increased over time from 2014 to 2018, with a 20% increase across the study period (OR = 1.2; 95% CI 1.1–1.2). The incidence rates per 100,000 population were 0.49 in 2014, 0.63 in 2015, 0.91 in 2016, and 1.05 in 2018. GeneXpert machine installation around the country also increased over time from 33 in 2014 to 249 machines in 2018**.**

### Trends in incidence of rifampicin-resistant tuberculosis among new and previously-treated patients

**Fig. 3:**
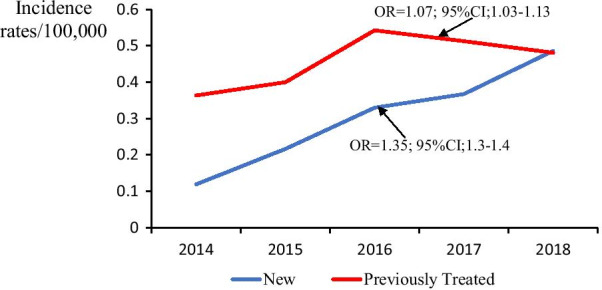
Trends of rifampicin resistant tuberculosis rates among new and previously treated cases, Uganda, 2014–2018

The incidence rates of RR-TB among both new and previously-treated cases increased from 2014 to 2018. Patients with previous TB treatment showed a greater incidence compared to the new cases over the entire study period. There was a bigger increase in the incidence among new TB cases (OR = 1.35; 95% CI 1.3–1.4) compared to previously-treated TB cases (OR = 1.07; 95% CI 1.03–1.13) (Fig. 3).

### Trends in incidence of different drug resistance types among patients with rifampicin-resistant tuberculosis

The incidence of multidrug resistance was persistently higher than the other types of drug resistance, with an overall incidence of 1.3 per 100,000 population over the 5 years **(**Additional file 1: Figure S1). Changes in incidence over time were not significant for any of the types of drug resistance over the study period except pre-XDR TB (OR = 1.36; 95% CI 1.09–1.7). Among patients with MDR-TB, there was an overall increase in incidence of 5% while among patients with mono-resistance, there was a 4% increase. In addition, there was an 8% decrease among patients with poly-resistance, and a 1% increase among patients with XDR. The pre-XDR TB resistance patients had the largest increase, at 36% (Additional file 1: Table S2).

## Discussion

The study shows an increase in incidence of rifampicin-resistant TB in Uganda during 2014–2018. Although the majority of patients with RR-TB had been previously treated, the increase in incidence was greater among new cases than among previously treated cases. The coverage of GeneXpert machines greatly improved during the study period, and this might have contributed to the improved detection for rifampicin resistance in most parts of Uganda.

This study found an overall incidence of RR-TB of 3.8 per 100,000 population in Uganda. This is fairly comparable with the 2018 WHO estimate of RR-TB incidence for the country of 3.5 per 100,000 [4]. The largest proportion of patients with RR-TB had MDR-TB, with resistance to streptomycin, isoniazid, rifampicin, and ethambutol. The TB National guidelines have evolved from resistance testing largely targeting retreatment cases only in 2011 to resistance testing for all TB patients with RR-TB regardless of prior TB history in 2016 [25, 26]. Our observation that previously-treated TB patients were more likely than new cases to have MDR-TB has been made in multiple other studies [27–29]. Such findings call for attention to treatment completion rates for new cases and extra vigilance for drug resistance monitoring among those with history of previous treatment for TB. In addition, these findings are a good basis for strict implementation of infection control practices to prevent transmission and emergence of new cases, including drug-resistant cases.

Although the incidence of XDR-TB was low and no significant change was observed during the study period, pre-XDR TB showed a significant increase in incidence. This level of second-line resistance is a cause for concern, especially in resource-limited countries such as Uganda. This has the potential of introducing case management repercussions such as adjustment of treatment regimens, need for new therapeutic agents and introduction of new rapid diagnostic tools. Data from previous studies suggest that a similar increase is being observed worldwide [30–34]. Such trends point to the need to expand laboratory capacity for culture and DST for both first and second line anti-TB drugs in TB endemic countries like Uganda. Currently, this capacity is limited to two public health laboratories; decentralization of laboratory services with improved specimen collection and transport for culture and DST TB is needed in the country. Early identification of pre-XDR TB patients would enable closer monitoring to prevent the progression to XDR-TB. The distribution of GeneXpert machines in Uganda has greatly improved which has contributed to the TB case detection. However, even with specimen referral system a17% GeneXpert coverage is still below the desired target for the population in Uganda.

Males and persons aged 25–44 had the highest incidence rates of RR-TB. Male predominance in active TB is widely-reported globally and has been previously reported in Uganda [35, 36]. Persons aged 25–54 have also been identified as the most-affected age group in other countries [37]. Males in this age group have been reported to undertake occupations that are associated with an elevated risk for TB [36, 38]. However, our findings contradict other studies elsewhere which have found more females than males with MDR-TB [39]. Despite such contradictions, we think that targeted screenings for persons aged 25–54 may help reduce the overall burden of MDR-TB, as well as the burden in this age group.

Northern Uganda, with widespread poverty [40] and poor living conditions in overcrowded communities, has been reported previously to have a high prevalence of TB [14, 26]. The northern region, especially the Karamoja region, has been reported to have TB treatment success rates below 72%, mostly attributed to the loss to follow up (LTFU) [14]. Studies have shown that patients who are LTFU are at higher risk of developing MDR-TB, compared to those who are not LTFU [41]. Therefore, programs that can enhance treatment follow-up are suitable for such regions.

The HIV prevalence in the north-central region is also among the highest in the country, at 7.2% compared to the national prevalence of 6.2%, while in the northeast it is 5.3% [42]. HIV co-infection is known to increase the risk for developing drug-resistant TB as mechanisms linking drug-resistant TB to HIV infection have been suggested. Drug malabsorption in HIV-infected patients, especially for rifampin and ethambutol, can also lead to drug resistance as the patient receives a subclinical dose [43–46]. Similarly, Kampala, with an HIV prevalence of 6.9%, had higher incidence rates of drug-resistant TB compared to other districts [42]. Multiple studies have shown that there is an association between drug-resistant TB and HIV [43], although at least one study in Uganda found the opposite [47]. Extra efforts are needed to control tuberculosis transmission in the different parts of the country after having understood each area’s specific risk factors.

## Study limitations

Our findings are based on routinely-generated laboratory surveillance data. This has the potential of underestimating the true burden of RR-TB because some patients do not access the healthcare system. Besides, RR-TB is only detected by GeneXpert which has a coverage of about 17% of all the TB diagnostic units in the country. This may have limited our ability to accurately determine the true incidence, and therefore the findings should be interpreted in this context. However, these findings provide a good reflection of the general trends in RR-TB incidence in the country over the study period.

## Conclusion

Rifampicin-resistant TB incidence rates have consistently risen throughout the last 5 years in Uganda, but the burden is regionally diverse. There has been a significant increase in new patients diagnosed with RR-TB, with most being among those previously treated. Males and persons in the age-group 24–54 years were more affected by drug-resistant TB than females and the other age-groups. Strengthening prevention and control programs, especially among the most affected sub-populations, is crucial to the goal of minimizing the burden of resistant tuberculosis especially in resource limited settings.

## Recommendations

It is important that all TB samples from patients with RR detected by the GeneXpert are referred for culture and DST before treatment initiation and contact tracing is initiated. It may also be efficient to adopt rapid diagnostic tests such as Cepheid Xpert MTB/XDR once they are approved in order to support the decentralization of DST testing. The National TB program should also consider having more machines installed in districts to enhance accessibility as well as instituting mechanisms to improve sample referral in areas without GeneXpert. Lastly, there is need to conduct a national TB drug resistance survey to determine the actual burden and risk factors associated with TB drug resistance in Uganda, and similar settings.

## Supplementary Information

**Additional file 1**: **Table S1**. Districts with highest RR TB incidence rates per 100,000 population, Uganda, 2014–2018. **Figure S1.** Trends of anti-tuberculosis drug resistance types among patients with rifampicin resistant tuberculosis, Uganda 2014–2018. **Table S2.** Odds ratios for changes in incidence by drug resistance types among RR-TB patients in Uganda 2014–2018.

## Data Availability

For confidentiality reasons the datasets are not publicly available. However, the data sets can be availed upon reasonable request from the corresponding author and with permission from the Uganda Ministry of Health.

## Abbreviations

IR: Incidence Rate
RR-TB: Rifampicin Resistant TB
MDR: Multidrug Resistance
XDR: Extensively Drug Resistance
TB: Tuberculosis
QGIS: Quantum Geographic Information System software
DST: Drug sensitivity testing
HIV: Human immune deficiency Virus
INH: Isoniazid
NTRL: National tuberculosis reference laboratory
RMP: Rifampicin
SM: Streptomycin
EMB: Ethambutol
PZ: Pyrazinamide
KAN: Kanamycin
LEV: Levofloxacin
MOX: Moxifloxacin
OFL: Ofloxacin
CAP: Capreomycin
AMK: Amikacin
WHO: World Health Organization
