# Supplementary material for: Epidemiological profile of patients with rifampicin-resistant tuberculosis: an analysis of the Uganda National Tuberculosis Reference Laboratory Surveillance Data, 2014–2018
Source: Antimicrob Resist Infect Control. 2021 May 8;10:76. doi: 10.1186/s13756-021-00947-2 (PMC8106164; doi:10.1186/s13756-021-00947-2)
Supplement: Supplementary file 1 — Additional file 1: Table S1. Districts with highest RR TB incidence rates per 100,000 population, Uganda, 2014–2018. Figure S1. Trends of anti-tuberculosis drug resistance types among patients with rifampicin resistant tuberculosis, Uganda 2014–2018. Table S2. Odds ratios for changes in incidence by drug resistance types among RR-TB patients in Uganda 2014–2018. [file 13756_2021_947_MOESM1_ESM.pdf]

Additional file 1

Supplementary Table S1: Districts with highest RR TB incidence rates per 100,000 population, Uganda, 2014-2018

Year	District with highest IR	IR/100,000
2014	Kitgum	6.4
	Lira	3.4
	Otuke	2.9
2015	Lira	7.4
	Kitgum	4.8
	Gulu	4.3
	Kampala	4.2
2016	Nakapiripirit	6.3
	Apac	5.5
	Kitgum	5.2
2017	Nakapiripirit	11
	Gulu	4.7
	Kitgum	4.2
	Napak	4
2018	Nakapiripirit	5.8
	Hoima	4.3
	Lira	3.9
	Napak	3.9

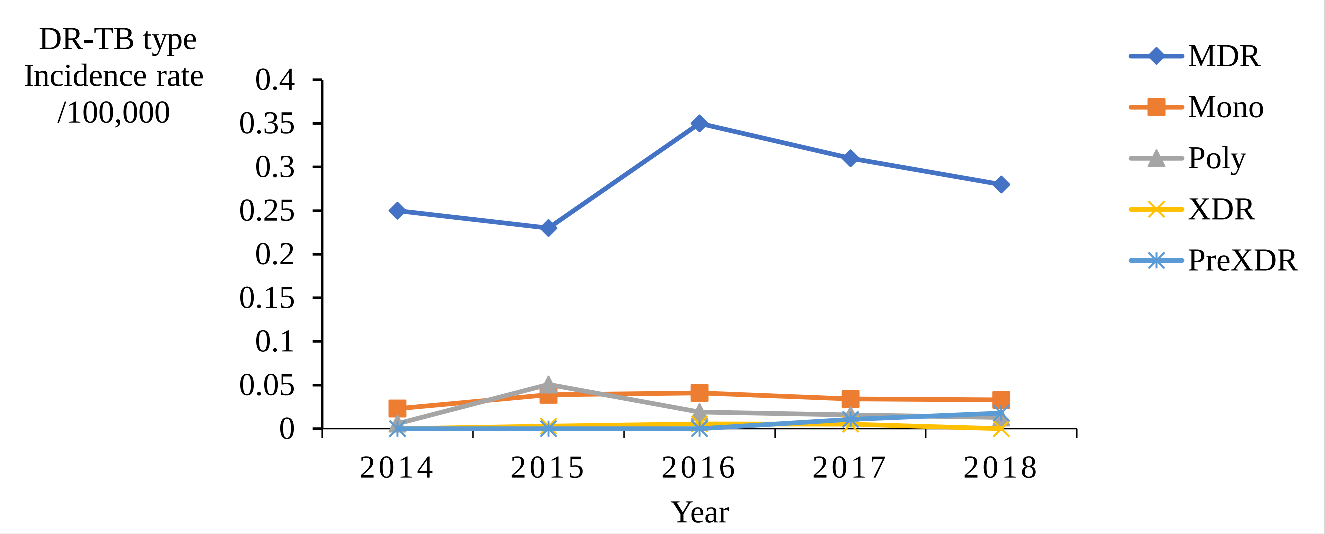

Supplementary Figure S1: Trends of anti-tuberculosis drug resistance types among patients with rifampicin resistant tuberculosis, Uganda 2014-2018

Supplementary Table S2: Odds ratios for changes in incidence by drug resistance types among RR-TB patients in Uganda 2014-2018

Drug Resistance Type	OR	95%CI
Mono resistance	1.04	0.89-1.2
Poly resistance	0.92	0.78-1.09
Multi drug resistance	1.05	0.99-1.1
Pre-XDR	1.36	1.09-1.7*
XDR	1.01	0.82-1.2

*Statistically significant at p=0.05
